# Supplementary material for: Genome-Wide Identification and Expression Analysis of YTH Gene Family for Abiotic Stress Regulation in Camellia chekiangoleosa
Source: Int J Mol Sci. 2024 Apr 3;25(7):3996. doi: 10.3390/ijms25073996 (PMC11012946; doi:10.3390/ijms25073996)
Supplement: Supplementary file 1 [file ijms-25-03996-s001.zip › ijms-2925837-supplementary.pdf]

Table S1. Sequences in the evolutionary tree.

Species name	Accession number	name code	sequnenceYTH motif
Mimulus guttatus	Migut.N00538.1	MgDF1A	KLSSVAPDREQYNQPEFSVTYDDARFFIIKSYSEDDVHKSIKYNVWASTPNGNKKLDAAYQDAQQRPGGCPVFLFFSVNTSGQFVGVAEMTGPVDFNKNVEYWQQDKWVGCFPVKWHIVKDLPNSLLKHITLENNENKPVTNSRDTQEVKLDQGLQVLKIFKDHTSKQCILDDFEFYEDRQKRIQEKKAKQQQY
	Migut.D02575.1	MgDF2A	EKSSVTPDLEEYNRPDFPETYSDAKFFIIKSYSEDDVHKSIKYNIWASTSNGNKKLEAAYQDAQQKPGGCPVFLFFSVNTSGQFVGVAEMVGPVDFNKNVEYWQQDKWIGCFPLKWHIVKDLPNSSLKHITLENNENKPVTNSRDTQEVKLEPGLAVLKIFKDHTSRQCILDDFDFYEDRQKKIQEKKARQQQF
	Migut.J00855.1	MgDF1B	VESTSKLHVHHLNSPDFITDYDRAKFFVIKSFSEDNIHKSIKYSVWASTPLGNRKLDAAYCETKEMEGICPVFLLFSVNASGQFCGVAEMIGPVDFENDADYWQQDRWSGQFPVKWHIIKDVPNSRFRHILLENNENKPVTHSRDSQEVKLEQGIEMLQIFKNHDAETSLLDDFNFYDEREKTLLERKAKQRAI
	Migut.O00389.1	MgDF1C	AQGNITIFKDQYNKIDFPVDYANAKFFVIKSYSEDDVHKSIKYNVWSSTPNGNKKLTTAYQDAHRTTLGDLRGCPIFLFFSVNASGQFCGVAEMTGPVDFHRDMDFWQQDKWSGSFPVKWHIIKDLPNSNFRHIILENNENKPVTNSRDTQEICYEKGLEMLRIYKSHTLKTSLLDDFMYYENRQRILQEERTRLLRK
	Migut.G00015.1	MgDF2C	AQGNITIFKDQYNKIDFPVDYANAKFFVIKSYSEDDVHKSIKYNVWSSTPNGNKKLTTAYQDAHRTGLGDLRGCPIFLFFSVNASGQFCGVAEMTGPVDFHRDMDFWQQDKWSGSFPVKWHIIKDLPNSNFRHIILENNENKPVTNSRDTQEICYKKGLEMLRIYKSHTLKTSLLDDFMYYENRQRILQEERTRLLRK
	Migut.N00839.1	MgDF3C	EQIGLPAKVDKFNLQDFQTVYENAKFYVIKSYSEDDIHKSIKYDVWSSTQNGNRKLDAAFNEANARTNCPVFLFFSVNASGQFVGVAEMIGHVDFSKSMDFWQLDKWNGFFPLQWHIVKDVPNTLLRHIILENNDNRPVTFTRDTQEIGLKQGLEMLSIFKNHSGKKSLLDDYSFYEKREKELKAKRKNAARS
	Migut.H01337.1	MgDC1A	SRNATPLPQGASRYFVVKSCNRENLELSVQQGVWATQRSNEAKLNEAFESVENIILIFSVNKTRHFQGCAKMTSRIGGSVGGGNWKHAHGTAHYGRNFALKWLKLCELTFDKTRHLRNPYNENLPVKISRDCQELEPSIGEQLASLLYLEPDSDLMAIAIAAELKREEEK
	Migut.M01886.1	MgDC2A	SRTATPLPQGTSRYFVVKSCNNENLELSVQQGVWATQRSNEAKLNEAFESVDNIILIFSVNKTRHFQGCAKMTSRIGGSISGGNWKNAHGTAHYGQNFSVKWLKLGELSFNKTRHLRNPFNENLPVKISRDCQELEPSIGEQLASLLYLEPDSDLMAVALAAEAKREEEK
	Migut.A00893.1	MgDC1B	SSEMKKDKLNNTRYFIIKSLNHENIQLSVKKEIWATQVMNEPILEEAFQNSGKVILIFSVNMSGFFQGYAQMMSSVGWRRDTIWSQASGKSNPWGRSFKVKWLRLNDLAFQKTLHLKNPWNDFKPVKISRDCQELPGDIGRALCELLDEGGGIDLNLKRDEIARGDFSASR
Eucalyptus grandis	Eucgr.B03590.1	EgDF1A	NKVSVIPDREQYNKADFPEDYEDAKFFIIKSYSEDDVHKSIKYSVWASTPNGNKKLDAAYREAQEKPGSSPVFLFFSVNTSGQFVGLAEMVGPVDFHKSVEYWQQDKWNGCFPVKWHIVKDVPNSMLKHITLENNENKPVTNSRDTQEVKLVQGLKMIKIFKEHPVKTCLFDDFEFYENRQKAIQEKKARQQYQ
	Eucgr.B03591.1	EgDF2A	NRSSVVPDCTQYNKVDFPEDYNDAKFFIIKSYSEDDVHKSIKYNVWASTPNGNKKLDAAYREAQEKAAGCPIFLFFSVNTSGQFVGLAEMVGPVDFHKNVEYWQQDKWMGCFPVKWHIVKDVPNNLLKHITLENNENKPVTNSRDTQEVKLPQGLKMIKIFQTHLVKTCLLDDFDFYENRQRAIQEKKARQLQF
	Eucgr.J03195.1	EgDF1B	SASITKIPHGSYNHIDFITEYDDAKFFVIKSYSEDNVHKSIKYGVWASTPNGNRKLDAAYREAKEKPDTCPVFLFFSVNASAHFCGVAEMVGPVDFDRSFDYWQQDRWSGQFPVKWHVIKDVPNSQLRHIVLENNDNKPVTNSRDTQEVKLEQGIEMLNIFKNYETDTSILDDFEFYEEREKVIQEGKARQQAS
	Eucgr.H04096.1	EgDF1C	AQGIIIIDTDQYNKDDFCVDYEDAKFFVIKSYSEDDVHKSIKYNVWSSTPHGNKKLQSAYEDAQKIAAEKTTNCPIFLFFSVNASGQFCGVAEMVGQVDFHKNMDFWQQDKWTGSFPVKWHIIKDVQNTAFRHIILENNENKPVTNSRDTQEIMQKQGLEMLKIFKNNGLKTSLLDDFTYYEGRQKLMQEERARMYVS
	Eucgr.E02496.1	EgDC1A	GRGPTPLPPGVSRYFIVKSCNRENLELSVQQGVWATQRSNEAKLNEAFDSAANVILIFSVNRTRHFQGCAKMTSRIGGAASGGNWKYAHGTAHYGRNFSVKWLKLCELSFQKTRHLRNPYNENLPVKISRDCQELEPSVGEQLASLLYLEPDSELMPISLAAESKREEEK
	Eucgr.D01949.1	EgDC1B	SIANRKGRWYSIRYFIIKSLNHHNLQLSIEKGIWATQVMNEPILEEAFHNSGKVILIFSVNMSGFFQGYAQMMSPIGWRRDKVWSQGNGRNNPWGRSFKVKWLRLYDLPFQKTLHLKNLLNDNKPVKISRDCQELPQDVGEALCELLDCDADVDGETRRDSAPLEK
	Glyma.08G124600.1	GmDF1A	DKTSTVPDRDQYNKADFPEEYTDAKFFVIKSYSEDDIHKSIKYNVWASTQNGNKKLDAAYHEAQQKPGGCPVFLFFSVNTSGQFVGLAEMIGPVDFNKSVEYWQQDKWNGCFPLKWHVVKDVPNNLLRHITLDNNENKPVTNSRDTQEVMLEPGLKLIKIFKEYTSKTCILDDFGFYEARQKTILEKKAKQQFP
	Glyma.05G166600.1	GmDF2A	DKTSTILECDQYNKADFPEEYTDAKFFVIKSYSEDDIHKSIKYNVWASTQNGNKKLDAAYQEAQQKPGGTPVFLFFSVNTSGQFVGLAEMIGPVDFNKSVEYWQQDKWNGCFPLKWHIVKDVPNNLLRHITLDNNENKPVTNSRDTQEVMLEPGLKLIKIFKEYTSKTCILDDFGFYEARQKTILEKKAKQQFP
	Glyma.19G110800.1	GmDF3A	KEVPPVPDKEQYNGKDFAENYSDAKFFVIKSYSEDDIHKSIKYSAWASTPNGNKKLDAAYQEAKEKPGGCPIFLLFSVNTSGQFVGLAEMLGPVDFGKTVDYWQQDRWTGCFSVKWHVIKDIPNSVLRHITLENNENKPVTNSRDTQEVKFEKGVQIAKIFKEHSSQTCILDDFGFYEAREKATQEKKSKEQQL
	Glyma.16G041600.1	GmDF4A	KEVPLVPDKEQYNGKDLAENYSDAKFFVIKSYSEDDIHKSIKYSAWASTPNGNKKLDSAYQEAKEKPGGCPIFLLFSVNTSGQFVGLAEMLGPVDFGKTVDYWQQDRWTGCFSVKWHVIKDIPNSVLRHITLENNENKPVTNSRDTQEVKFEKGVQLVKIFKEHSSQTCILDDFGFYEDREKVTQEKKSKEQQL
	Glyma.02	GmDF5	KEVPLVPNNEQYNGKDFPENYSDAKFFVIKSYSEDDIHKSIKYKVWASTFNGNKKLDAAYHESKEKPG

	G072000.1	A	DCPVFLLFSVNTSGQFVGLAEMVSPLDFGRTVEYWQQDRWSGCFSVKWHIIKDIPNSVLRPITLENNENKPVTNSRDTQEVKFEKGIQILKIFKQHSSKTCILDDFGFYETREKMIQERKSMEQQL
	Glyma.08G226100.1	GmDF1B	SASTAKFQNESLNRSDFATDYKDAKFFVIKSYSEDNVHKSIKYGVWASTPNGNRKLDDAYRQAMEKQDACPIFLFFSVNASAQFCGVAEMVGPVNFDKSVDFWQQDKWSGQFPLKWHIIKDVPNSQFRHIILENNDNKPVTNSRDTQEVKLGQGVEMLTIFKNYETDVSILDDFDFYEDRQKAMQERKARQQSS
Glycine max	Glyma.07G000900.1	GmDF2B	SGSTAKFQNESLNWSDFATDYKDAKFFVIKSYSEDNVHKSIKYGVWASTPNGNRKLDAAYLQAMEKQDACPIFLFFSVNASAQFCGVAEMVGPVNFDKSVDFWQQDKWSGQFPVKWHIIKDVPNSQFRHIVLENNDNKPVTNSRDTQEVKLTQGVEMLTIFKNYETDVSILDDFDFYEDRQKAMQERKARQQSS
	Glyma.17G038400.1	GmDF1C	TQENIIISTDQYNREDFPVNYENAKFFVIKSYSEDDVHKSIKYNVWSSTPHGNKKLQSTHEDAKRIASGNFGSCPIFLFFSVNASGQFCGVAEMIGPVDFNKDMDFWQQDKWSGSFPVKWHIIKDVPNANFRHIILENNENKPVTNSRDTQEIMYSKGLEMLKIFKNHNLKTSLLDDFMYYENRQKIMQEEKAKLLIR
	Glyma.07G233400.1	GmDF2C	TQENIIISTDQYNREDFPVNYENAKFFVIKSYSEDDVHKSIKYNVWSSTPHGNKKLQSAHEDAKRIASGKFGSCPIFLFFSVNASGQFCGVAEMIGPVDFNKDMDFWQQDKWSGSFPVKWYIIKDVSNANFRHIILENNENKPVTNSRDTQEIMYSKGLEMLKIFKNHHLKTSLLDDFIYYENRQKIMLEEKTKLLIR
	Glyma.15G136400.1	GmDF3C	EQGNIVIYPDQYNMEDFPLGYENAKFFVIKSYSEDDVHKSIKYNVWSSTPHGNKKLENAYEDAKKIAAEKSEVCPIFLLFSVNASGQFCGVAEMVGTVDFSKNMDFWQQDKWSGSFPVKWHIIKDVPNPNFRHIILENNENKPVTNSRDAQEIMYLKGLEMLKIFKNHTLKTSLLDDFMYYENRQKIMQDEKAKFLVK
	Glyma.09G031200.1	GmDF4C	EQGNIVIYPDQYNKEDFSLDYENAKFFVIKSYSEDDVHKSIKYNVWSSTPHGNKLENAYEDAKKIAAEKSEVCPIFLFFSVNASGQFCGVAEMVGTVDFNKNMDFWQQDKWSGSFPLKWHIIKDVPNPNFRHITLENNENKPVTNSRDTQEIMYWKGLEMLKIFKNNTLKTSLLDDFMYYENRQKIMQDEKA
	Glyma.15G265400.1	GmDF5C	GNVTNKIRADQYNLPDFPTKYDHALFFVIKSYSEDDIHKSIKYNVWASTPNGNKRLDGAFQDAQKRMEEKGCKCPVFLFFSVNASGQFCGVAEMTGRVDFNKSMDFWQQDKWNGYFPVKWHIIKDVPNPQLRHIILENNDHKPVTSSRDTQEVSFPQGVEMLNIFKNYVARTSILDDFEFYESRQKVMQEKKTRQSMP
	Glyma.08G162200.1	GmDF6C	GNVTNKIRTDQYNLPDFPTKYDHALFFVIKSYSEDDIHKSIKYNVWASTPNGNKRLDGAFQDAQKRMEEKGCKCPVFLFFSVNASGQFCGVAEMTGRVDFNKSMDFWQQDKWNGYFSVKWHIIKDVPNPQLRHIILENNDHKPVTNSRDTQEVSFPQGVEMLNIFKNYVARTSILDDFEFYESRQKVLQEKKTRQSMP
	Glyma.15G128500.1	GmDC1A	NRTATPLPQGISRYFIVKSCNRENLELSVQQGVWATQRSNESKLNEAFDSVENVILVFSVNRTRHFQGCAKMTSRIGGSVAGGNWKYAHGTAHYGRNFSVKWLKLCELSFHKTRHLRNPYNENLPVKISRDCQELEPSIGEQLASLLYLEPDSELMAISVAAESKREEEK
	Glyma.09G022200.1	GmDC2A	NRTATPLPQGISRYFIVKSCNRENLELSVQQGVWATQRSNESKLNEAFDSVENVILIFSVNRTRHFQGCAKMTSKIGGSVAGGNWKYAHGTAHYGRNFSVKWLKLCELSFHKTRHLRNPYNENLPVKISRDCQELEPSIGEQLASLLYLEPDSELMAISVAAESKREEEK
	Glyma.01G214100.1	GmDC1B	PTGIGVEKWHNIKYFIIKSLNHQNIHLSIEKGIWATQIMNEPILEEAFHNSGSVILIFSVNMSGSFQGYAQMMSSIGRGRDNVWSEGTGKSNPWGRSFKVKWMCLNDLPFHKTLHLKNPLNDYKPVKISRDCQELSPDIGLALCELLDGKNYTDCLPTSLSRDEFSLKG
	Glyma.11G027800.1	GmDC2B	PAGIGVEKCHNTKYFVIKSLNHQNIHLSIEKGIWATQIMNEPILQEAYHNSGSVILIFSVNMSGSFQGYAQMMTSIGRGRDNAWSEGTGKSNPWGRSFKVKWLCLNDLPFHKTLHLKNPLNDYKPVKISRDCQELSPDIGLALCKLLDGKNDTDGLLTSLSRDEFSLKG
	Prupe.6G002200.1	PpDF1A	KDKTSVPDREQYNKADFPEDYTDAKFFIIKSYSEDDVHKSIKYNVWASTPNGNKKLHAAYQEAQEKSGGCPVFLLFSVNTSGQFVGLAEMLGPVDFNKNLEYWQQDKWNGCFPVKWHIVKDVPNSLLKHITLENNENKPVTNSRDTQEVKLEPGLKMIKIFKEHLSKTCILDDFGFYESRQKTIQEKKAKQQQF
Prunus persica	Prupe.3G027100.1	PpDF1B	NTTATKVHDESYNKPDFVTEYNDAKFFIIKSYSEDNVHKSIKYGVWASTPNGNRKLDAAYREVKEKHDGCPIFLFFSVNASAQFCGVAEMVGPVDFDKSLDYWQQDKWSGQFPVKWHMIKDVPNSQFRHIILENNDNKPVTNSRDTQEVKLEHGIEMLNIFKNYETDMSILDDFDFYEDRQKAMQERKARQQAS
	Prupe.1G002400.1	PpDF2B	GLSASGINLDSFNRLDFVTDYEDAKFFIIKSFSEDNVHKSIKYSVWASTPHGNKKLDAAYHEAQRIKGSCPVFLFFSVNASGQFCGVAEMVGSVDFEKDADYWQQDRWSGQFPVQWHIIKDVPNIRFRHILLENNDNKPVTHSRDCQEVNLKQGIELLKIFGDYDARTSIIDDFEFYDEREKSLKERKVRQQAC
	Prupe.1G109700.1	PpDF1C	AQGNIIIRTDQYNKDELPVDYVDAKFFVIKSYSEDDVHKSIKYNVWSSTPHGNKKLNSAYEDALRIAAGKPRGCPIFLFFSVNASGQFCGVAEMGGPVDFNKDMDFWQQDKWSGSFPVKWHIIKDVPNTSFRHIVLENNENKPVTNSRDTQEIMYKKGLEMLKIFKNHTLKTSLLDDFMYYEERQKFMQEERSRLLVR
	Prupe.4G124900.1	PpDF2C	DSIASAIRKDQYNLPDFPTKYEQALFFVIKSYSEDDIHKSIKYNVWASTPNGNKRLDSAYQDAEEKVAQKSCKCPVFLFFSVNASGQFCGVAEMIGRVDFNKSMDFWQQDKWNGYFPVKWHIIKDVPNPQLRHIILENNENKPVTNSRDTQEVKFLQGIEMLNIFKNHLSKTSILDDFDFYESRQKVMQEKRIRQSTP
	Prupe.1G579100.1	PpDF3C	EELSFTVHRDQYNLPDFQTDYEKAKFYVIKSYSEDDVHKSIKYDVWASTPNGNKKLDASFRDAESKSRETGTQCPIFLFFSVNGSGQFIGLAEMAGQVDFNKDMDFWQVDKWSGFFPVKWHVIKDIPNTQLRHIILENNDNRPVTFTRDTQEIGLKQGLEMLNIFKSYTAKTSLLDDFIFYEDREKSLKAKRSSKPAT
	Prupe.4G089500.1	PpDC1A	ANRSAPLPQGISRYFIVKSCNRENLELSVQQGVWATQRSNESKLNEAFDSAENVILIFSVNRTRHFQGCAKMMSRIGGSVSGGNWKYAHGSAHYGRNFSVKWLKLCELSFHKTRHLRNPYNENLPVKISRDCQELEPSIGEQLASLLYLEPDSELMAVSIAAESKREEEK
	Prupe.5G086300.1	PpDC1B	TTGRKKGTLYDTRYFIIKSLNHQNIQLSIEKGIWATQVMNEPILEEAFHNSGKVILIFSVNMSGSFQGYAQMMSSVGWRRDNVWSQGSSRSNPWGRSFKVKWLQLNDLPFQKTLHLKNPLNEYKPVKISRDCQELSPDVGEALCELLDGTNDVNGLNSVAMEDFPSKR

Populus trichocarpa	Potri.003<br>G222700.1	PtDF1A	DKTTVVPDREQYNKADFPEEYDNAKFFIIKSYSEDDVHKCIKYNVWASTPNGNKKLDAAYQEAEQKS<br>GGCPVFLLFSVNTSGQFVGLAEMTGRVDFDKSVEYWQQDKWTGYFPVKWHIVKDVPNSFLKHITLEN<br>NENKPVTNSRDTQEVKLEQGLKLIKIFKDHSSKTCILDDFVFYEDREKMIQEKKAKQQQL
	Potri.001<br>G002000.1	PtDF2A	DKTTEVPDREQYNKADFPVEYVDAKFFIIKSYSEDDVHKCIKYNVWASTPNGNKKLDAAYQEAGQKS<br>GGCPVFLLFSVNTSGQFVGLAEMTGRVDFDKSVEYWQQDKWTGYFPVKWHFVKDVPNSLLKHITLEN<br>NENKPVTNSRDTQEVKLEQGLEMIKIFKEHSSKTCILDDFGFYEDREKMIQEKKAKQQQL
	Potri.019<br>G034300.1	PtDF3A	ENLLQIPDREQYNKEDFPEEYSDAKFFVIKSFSEDDVHKSIKYSVWTSTPNGNKKLDAAYKQGKENPGD<br>CPVFLLFSVNTSGQFVGLAEMVGPVDFNKTVEYWQQDKWTGCFPLKWHIIKDVPNGCLRHITLENNE<br>NKPVTNSRDTQEVIFEKGVQILKIFKDHKGKTSILDDFSFYAGRERIMQEKRAKHNIH
	Potri.014<br>G001000.1	PtDF1B	SKPSAKIHDESYNQSDFVTEYKDAKFFIIKSYSEDNVHKSIKYGVWASTPNGNRKLDTAYHEAKDKQDP<br>CPVFLLFSVNASAQFCGVAEMVGPVDFDKSVDYWQQDKWSGQFPVKWHIIKDVPNSQFRHIVLENN<br>DNKPVTNSRDTQEVKLEQGVEMLNIFKNYETDTSILDDFDFYEDRQKAMQDRKARQQAS
	Potri.007<br>G002800.1	PtDF2B	SKPSAKIHDESYNQPDFVIEYKDAKFFIIKSYSEDNVHKSIKYGVWASTPNGNRKLDTTYREAKEKQDPC<br>PVFLLFSVNASAQFCGVAEMTGPVDFDKSVDYWQQDKWSGQFPVKWHIIKDVPNSQFRHIVLENND<br>NKPVTNSRDTQEVKLEQGIEMLNIFKNYETDMSIIDDFDFYEDRQKAMQERKARQQAS
	Potri.006<br>G079900.1	PtDF3B	NSASSGIQLDLYNQLDFVTDYKDAKFFIIKSFSEDNVHKSIKYSVWASTPHGNKKIDAAYREAKEKEGN<br>CPVFLLFSVNASGQFCGVAEMVGPVDFEKDAEYWQQDRWNGQFPVQWHIVKDVPNSRFRHILLENN<br>DNKPATHSRDSQEVKLEQGIEMLKIFKDHDAPTSILDDFDFYDQCERALKERKAKQQPS
	Potri.018<br>G149800.1	PtDF4B	NSASSGVQLDLYNRPVFVTDYKNAKFFIIKSFSEDNVHKSIKYSIWASTPHGNKKIDAAYREAKEKEGN<br>CPVFLLFSVNASGQFCGVAEMVGPVDFEKDADYWQQDRWNGQFPVQWHIIKDVPNSRFRHILLENN<br>DHKPVTHSRDSQEVKLEQGIEMLKIFKDYDAPTSIIDDFGFYDQCERALKERKAIQQPS
	Potri.001<br>G056100.1	PtDF5B	QKAVADVHSESHNQVDFATDYKDAKFFVIKSYSEDNVHKSIKYGVWASTPNGNKKLDAAYREAKEN<br>HGTCPIFLLFSVNASAQFCGVAEMVGPVDFDKNVDFWQQDKWSGQFPVKWHIIKDVPNSQFRHIVLE<br>NNDNKPVTNSRDTQEVELEQGAEMLGIFKNYESHSSILDDFQFYEERQKVMQVRKSRPQAS
	Potri.010<br>G152300.1	PtDF1C	GLGNIVIQTDQYNKDDFSTDYLDAKFFVIKSYSEDDVHKSIKYNVWSSTPHGNKKLQTAFEDAQKLAV<br>GRPRGCPIFLFFSVNASGQFCGVAEMIGPVDLHRDMDFWQQDKWSGSFLVKWHIIKDIPNSSFRHIILE<br>NNENKPVTNSRDTQEIMYKQGLEMLKTFKNHPLRTSILDDFMYYENRQKIMQDEKARLMFK
	Potri.008<br>G100200.1	PtDF2C	ELGNIVIQTDQYNKDDFSTDYADAKFFVIKSYSEDDVHKSIKYNVWSSTPHGNKKLHSAFEYAQKLDL<br>GRPRGCPIFLFFSVNASGQFCGVAEMVGPVDFNRDMDFWQQDKWSGSFLVKWHIIKDIPNSSFRHIILE<br>NNENKPVTNSRDTQEIMYKQGLEMLKMFKNHPLKTSILDDFVYYENRQKIMQEEKARLMFK
	Potri.010<br>G175500.1	PtDF3C	NSITSVISRDQYNLPDFPTNYDHAFFFVIKSYSEDDIHKSIKYNVWASTPNGNKRLNSAYLDSQQKIAQI<br>GCSCPVFLFFSVNASGQFCGVAEMTGRVDFNKNMDFWQQEKWNGYFPVKWHIIKDIPNPQLRHIILE<br>NNENKPVTNSRDTQEVKFPQGIEILNIFKNYVSKTSILDDFDFYESRQKVMHERRPRSLIS
	Potri.008<br>G080800.1	PtDF4C	NSIASVISRDQYNLPDFPTKYNHAFFFVIKSYSEDDIHKSIKYNVWASTPNGNKRLDSAYQDAQQKIAE<br>KGNSCPVFLFFSVNASGQFCGVAEMVGRVDFNKNMDFWQQDKWNGYFPVKWHIIKDVPNPQLRHII<br>LENNENKPVINSRDTQEVKFPQGIEILNIFKNYVTKTSILDDFDFYESRQKVMQEKRPRPFIP
	Potri.004<br>G223800.1	PtDF5C	DMLGFAMHKEQYNLPDFEIEYSNAKFFVIKSYNEDDIHKSIKYDVWASTPNGNKKLDAAFHNAEEVSS<br>ETGTKCPIFLFFSVNGSGQFVGLAEMVGQVDFNKDMDFWQIDKWNGFFPVKWHVIKDIPNGQLRHIV<br>LENNDGHSVTFSRDTQEIGLEKGLEMLNIFKSYSAKTSMLDDFNFYENREKSLNTKKSNKPAT
	Potri.003<br>G008400.1	PtDF6C	DELGIAMRKEQYNLPDFETEYANAKFFVIKSYSEDDIHKSIKYDVWASTPNGNKKLDAAFHNAEEVSS<br>DTGYKCPIFLFFSVNGSGQFVGFAEMVGQVDFNKDMDFWQIDKWNGFFPVKWHVVKDIPNGHLRHI<br>VLENNDGHSVTFSRDTQEIVLKQGLEMLNIFKSYSAKTSLLDDFNFYEKREKSLNTKKGNKPAT
	Potri.001<br>G357800.1	PtDC1<br>A	SSFFSCVSPSQFVYFIVKSCNRENLELSVQQGVWATQRSNEIKLNEALDSADNVILIFSVNRTRHFQGCA<br>KMASKIGASVGGGNWKYAHGTAHYGRNFSVKWLKLCELSFHKTRHLRNPFNENLPVKISRDCQELEP<br>SIGEQLASLLYLEPDSELMAVSLAAEAKREEEK
	Potri.011<br>G089800.1	PtDC2<br>A	VLYVVVFWLRVKLYFIVKSCNLENLELSVQQGVWATQRSNEPKLNEAFDSAENVILIFSVNRTRHFQGC<br>AKMTSKIGASVGGGNWKYAHGTAHYGRNFSVKWLKLCELSFHKTRHLRNPFNENLPVKISRDCQELE<br>PSIGEQLASLLYLEPDSDLMAISVAAEAKREEEK
	Potri.001<br>G113500.1	PtDC1B	SSINKKRKLCNTRYFIIKSLNQHNIQLSIENGIWATQVRNEPILEEAFHNSGRVILIYSVNMSGFFQGYAQ<br>MISSVGWRHDNLWSEGSGKSNPWGRSFKVKWLRLNDLPFQKTLHLKNPLNDYKPVKISRDCQELPED<br>IGEALCELIDGERDTDGMVKSFPRDDLPMKR
	GSVIVT0<br>102549400<br>1	VvDF1<br>A	DRTSVFPDRDQYNHADFPVEHTEAKFFIIKSYSEDDVHKSIKYNVWASTPNGNKKLDAAYREAQEKSA<br>GCPVFLFFSVNTSGQFVGLAEMVGHVDFHKNVEYWQQDKWNGCFSVKWHVVKDVPNSLLKHITLE<br>NNENKPVTNSRDTQEVKLEQGLQMLKIFKEHCSKTCILDDFAFYESRQKTIQEKKAKQQQF
	GSVIVT0<br>103339500<br>1	VvDF2<br>A	GNLTLVPDKEQYNSEDFPENYSDAKFFIIKSYSEDDVHKSIKYNMWASTANGNKKLDAAYQEAQGKS<br>GSCPIFLLFSVNASGQFVGVAEMVGSVDFNRSLEYWQQDKWTGCFPVKWHVIKDIPNSLLKHITLENN<br>ENKPVTNSRDTQEVKFEQGIQVLKIFKNHSSKTTILDDFGFYEARQRTMQEKKAKQQLF
	GSVIVT0<br>102946000<br>1	VvDF1<br>B	STSTAKTNDGLYNQLSFVTEYKDAKFFIIKSYSEDNVHKSIKYGVWASTPNGNRKLDAAYREAKEKQA<br>ACPVFLLFSVNASAQFCGVAEMVGPVDFDKSVDYWQQDKWSGQFPVKWHIIKDVPNSQFRHIVLEN<br>NDNKPVTNSRDTQEVKLEQGIEILNIFKNYETDTSILDDFDFYEDRQKAMQERKARQQAS
	GSVIVT0<br>101049400	VvDF2<br>B	NGTCTGVHRESYNRLDFVTEYKDAKFFVIKSYSEDNVHKSIKYGVWVSTPNGNKRLNSAFHEAKEKHG<br>NCPIFLLFSVNASAQFCGVAEMVGPVDYDKSVDYWKQDKWTGQFPVKWHIIKDVPNSQFRHIILENN

	1		DNKPVTNSRDTQEVELEQGNEMLNIFRNYESDSSILDDFDFYEDRQKAMQEKKTQHKAS
Vitis vinefera	GSVIVT01032236001	VvDF3B	DASNSVVHFDSYNQPDFVTDYENAKFFIIKSYSEDNVHRSIKYSVWASTALGNRKLDAAYQKAKEIETNCPIFLCFSVNASSQFCGVAEMVGPVNFEKDAEYWQQDRWSGLFPVKWHIIKDVPNSLFRHILLENNENKPVTHSRDTQEVNLEQGIEMLKIFKSYDAHTSILEDFDFYEQRERASKERKARQQAC
	GSVIVT01017649001	VvDF1C	AQGNIIIYTDQYNKDDFPVDYVDAKFFVIKSYSEDDVHKSIKYNVWSSTPNGNKKLENAYEDAQRIALGKRRGCPIFLFFSVNASGQFCGVAEMIGPVDFHKDMDFWQQDKWSGSFPVKWHIIKDVPNTNFRHIILENNENKPVTNSRDTQEIRFKQGLEMLKIFKNHTTKTSLLDDFMYYENRQRIMQDEKTRLLIK
	GSVIVT01036696001	VvDF2C	NCIASLIRRDQYNLPDFPTKYDHAFFFVIKSYSEDDIHKSIKYNVWASTPNGNKRLDGAYQEAKERMGDRGSKCPVFLFFSVNASGQFCGVAEMIGRVDFNKNMDFWQQDKWNGFFPVKWHIIKDVPNPQLRHIILENNDNKPVTNSRDTQEVRFPQGIEILNIFKNYVSKTSILDDFDFYESRQKVMQDKKIKPSMP
	GSVIVT01023248001	VvDF3C	EELGLMVRRDQYNLQDFQTEYENAKFYVIKSFSEDDIHKCIKYDVWASTPNGNKKLDAAFHDAEAKANETGTKFPIFLFFSVNGSGQFVGVAEMVGQVDFNKDMDFWQLDKWNGFFPVKWHIVKDIPNSQLRHITLESNENRSVTYTRDTQEIGLKQGVEMLKIFKNYSARTSMFDDFNFYENREKSLHARRSSKPPP
	XP_002281594.1	VvDC1A	NKTASPLPQGISRYFIVKSCNRENLELSVQQGVWATQRSNEAKLNEAFDSVENVILIFSVNRTRHFQGCAKMTSKIGGFVGGGNWKYAHGTAHYGRNFSVKWLKLCELSFHKTRHLRNPYNENLPVKISRDCQELEPSIGEQLASLLYLEPDSELMAISLAAESKREEEK
	GSVIVT01019895001	VvDC1B	SSANKKGKLYSTRYFIIKSLNHHNIQLSMEKGIWATQVMNEPILEEAFHNSGKVILIFSVNMSGFFQGYAQMMSSVGWRRDNVWSQGSGGNNPWGRSFKVKWLRLHDLPFQKTLHLKNPLNDYKPVKISRDCQELSQEIGEALCELLDGKTDVGDNLRKNDFSGDDLPPRR
	Solyc01g028860.2.1	SlDF1A	DKPSLMPDREQYNHPDFPVTYADAKFFIIKSYSEDDVHKSIKYNVWASTPNGNKKLDAAYQEAQQNSGGCPVFLFFSVNTSGQFVGVAEMVGPVDFNKNVEYWQQDKWVGCFPVKWHIVKDVPNSLLKHITLENNENKPVTNSRDTQEVKVEQGLQVLKIFKDHISKQCILDDFEFYEDRQKRIQDKKAKQQLF
	Solyc05g032850.2.1	SlDF1B	VELTSILQPGQYNRPEFVTDYEHAKFFVIKSFSEDNVHKSIKYGVWASTPQGNRKLDAAYAEAKEMNANCPVFLFFSVNASGQFCGVAEMVGPIDFENSAEYWQQDRWSGKFPVKWHVIKDVPNSQFRHILLEHNDNKPVTHSRDSQEVKLPEGLEMLKIFKNYETDTSLLDDFTYYDEREKSLLEKKSKQRPL
	Solyc12g099090.1.1	SlDF1C	VQGNIVIHADEYNRGDFLMDFVNAKFFVIKSYSEDDVHKSIKYNVWSSTPNGNKKLNSAYEDAQRITPGNPRGCPIFLFFSVNASGQFCGVAEMTGPVDFYKDMDFWQQDKWSGSFPVKWHFIKDVPNPNFRHIILENNENKPVTNSRDTQEIRYKKGIEMLKVFKDYASRTSLLDDFMYYENRQKLLQEEKAKLLIR
Sonalum lycopersicum	Solyc01g103540.2.1	SlDF2C	DQLVPAVQREKYNKEDFKTQYDNAKFYIIKSYSEDDIHKCVKYDVWSSTPNGNKKLDTAFVESEAKASGTGSSCPVFLFFSVNGSGQFLGVAEMVGQVDFNRNMDFWQLDKWSGFFPLKWHIVKDVPNTQFRHIILENNDNRPVTYSRDTQEIGLKEGLEMLNILKNYSEKTSILDDFNFYEKREKVLKAKRSSKPVI
	Solyc02g021760.2.1	SlDC1A	DRTAVVLPQGTSRYFIVKSCNRENLELSVQQGVWATQRSNEAKLNEAFDSVENVILIFSVNRTRHFQGCGKMTSRIGGAANGGNWKHEHGTAHYGRNFSLKWLKLCELSFQKTHHLRNPYNENLPVKISRDCQELEPSVGEQLASLLYLEPDSELMAISLAAESKRLEEK
	Solyc02g070240.2.1	SlDC2A	NQAAIPLPQGPSRYFIVKSCNRENLELSVQQGVWATQRSNEAKLNEAFDSVENVILIFSINRTRHFQGLAKMTSRIGGAAKGGNWKHEHGTAHYGRNFSVKWLKLCELSFQKTRHLRNPYNENLPVKISRDCQELEISVGEQLASLLYVEPDSELMAISLAAESKREEER
	Solyc08g007730.2.1	SlDC1B	STAIEKGKLYSIRYFVIKSLNHENIQLSVNRGIWATQAMNEAILDEAFHNSSKVILIFSVNTSGYFQGYAQMISSVGLRRDQVWSQGNGGRNSWGRSFEVNWLRLYDLPFQRTLHLKNPWNQNKPVKISRDCQELPPDIGEALCELLDGQDALDVNLKMDVFARNELSSKR
	Medtr8g088390	MtDF1A	EKNSTTPDREQYNKADFPEEYTDAKFFVIKSYSEDDIHKSIKYNVWASTQNGNKKLDAAYQEAQQKSGGCPIFLLFSVNTSGQFVGLAEMTGPVDFNKSLEYWQQDKWMGCFPLKWHIVKDVPNNVLRHITLENNENKPVTNSRDTQEIMLEPGLKLLKIFKEYSSKTCILDDFGFYEGRQKTILEKKAKQQFP
	Medtr7g085500	MtDF2A	EVVPLVLNKEQYNGEDLSENYSDAKFFIIKSYSEDDVHKSIKYSVWASTPNGNKKLDAAYQEAGGCPIFLLFSVNTSGQFVGLAEMTGPVDFDKTVEYWQQDRWTGCFNVKWHIIKDIPNGVLRHITLENNENKPVTNSRDTQEVKFEKGVQIVKIFKEHASKTSILDDFGFYESREKTTQERKFKEQQL
Medicago truncatula	Medtr6g032970	MtDF3A	KEVHPVPDKQQYTGEDLSENYSDAKFFVIKSYSEDDIHKSMKYNVWTSTPNGNKKLDAAYLEAKEKSADCPIFLLFSVNTSGQFVGLAEMVSPVDFDRTVEYWQQDRWTGCFSVKWRIIKDIPNNVLRHITLENNENKPVTNSRDTQEVKFEKGVEILKIFKEHSSKTCILDDFAFYEAREKTIQEKKAKEQLS
	Medtr4g005110	MtDF1B	NASTAKFQDESLNRPDFATDFKDAKFFVIKSYSEDNVHKSIKYGVWASTPNGNRKLDAAYCQAKEKQDACRIFLFFSVNASAQFCGVAEMVGPVNFDKSVDFWQQDKWSGQFPVKWHIIKDVPNSQFRHIVLENNDNKPVTNSRDTQEVKLQQGIEMLTIFKNYETDTSILDDFDFYEDRQKAMQERKARQQSS
	Medtr4g123630	MtDF1C	NTQENIIYTDQYNREDFPIDNEIAKFFVIKSYSEDDVHKSIKYNVWSSTPHGNKKLQSAYEDARRKATGKSGGCPIFLFFSVNASGQFCGVAEMVGPVDFNKDMDFWQQDKWSGSFPVKWHIIKDVPNGNFRHIILENNEFKPVTNSRDTQEIMYRKGLEMLKIFKNYTLKTSLLDDFIYYENRQKVMQEEKTKFLMK
	Medtr2g063460	MtDF2C	VNGNTKIRTDQYNLPDFPTKYDHALFFVIKSYSEDDVHKSIKYDVWASTPNGNKRLDNAFQDAQNRMEEKGSKCPVFLFFSVNASGQFCGVAEMIGRVDFNKSMDFWQQDKWNGYFPVKWHIIKDIPNPQLRHIILENNDHKPVTNSRDTQEIHFPQGIEMLNIFKNYVSRTSILDDFDFYESRQKVMQEKKIRQPMP
	Medtr2g032660	MtDF3C	EQDSIIIYTDQYNKEDFPLDYDNAKFFVIKSYSEDDVHKSIKYNVWSSTVHGNRKLGNAYEDTKKVSAEKSGVCPIFLFFSVNASGQFCGVAEMIGSVDFNKDMDFWQQDKWSGSFPVKWHIIKDVPNPNFRHIILDNNENKPVTNSRDTQEIMYSKGLEMLKIFKNHTLKTSLLDDFMYYENRQKIMHDQKAKLLIR

	Medtr2g005840.1	MtDF4C	DEFAFTVCRDQYNLPDFQTKYETAKFYMIKSFNEDDIHKGIKYDVWTSTPHGNKKLNAAFQNAEAKL<br>SQTGTQCPIFLFFSVNASGQFVGVAEMLGPVDFKKDMKFWKLDKYNGFFPIKWHIIKDVPNRQFAHIT<br>LQINENKCVTFSRDTQEITLKEGLEMLKIFKSYPAKTSLLDDFDFYENREKLCRSQRTEHTGW
	orange1.1g005158m	CsiDF1A	DKISLSPDRDEYNKADFPEEYTDAKFFVIKSYSEDDVHKSIKYSVWASTPNGNKKLDAAYQEAQQKSRS<br>CPVFLLFSVNTSGQFVGLAEMAGPVDFNKNVEYWQQDKWTGCFPVKWHIVKDVPNSLLKHITLENN<br>ENKPVTNSRDTQEIKLEQGLKLIKIFKDHPSKTCILDDFGFYETRQKTIQEKKAKQQQF
	orange1.1g010141m	CsiDF2A	DNLPLIPDKEKYSGEDFPESYSDAKFFIIKSYSEDDVHKSVKYNMWTSTPNGNKKLDAAYREAKEKSSD<br>CPVFLLFSVNASGQFVGVAEMVGPVDFDKTVEYWQQDKWVGCFPLKWLIIKDVPNSSLRHITLENNE<br>NKPVTNSRDTQEVNFEIGIQILKIFKSHSSKRCILDDFGFYEARERIMQQKKAKQHQL
	orange1.1g005934m	CsiDF1B	SKSVVKILDESYNQPDFVTDYKDAKFFIIKSYSEDNVHKSIKYGVWASTPNGNKKLDAAYCEAKEKQDP<br>CPVFLFFSVNASAQFCGVAEMIGPVDFEKSVDYWQQDKWSGQFPVKWHIIKDVPNSQFRHIVLENND<br>NKPVTNSRDTQEVKLEQGIEMLNIFKNYVTDMSILDDFDFYEDRQKAMQERKARQQAS
Citrus sinensis	orange1.1g038459m	CsiDF2B	NSSGSGINFELYNQFDFPTDYENAKFFVIKSFSEDNVHKSIKYGVWASTPHGNKKLDAAYHEAKETDN<br>RCPIFLLFSVNASGQFCGVAEMVGPVDFENSADYWQQDRWSGQFPVKWLIIKDVPNSRFRHLLLENN<br>DNKPVTHSRDSQEVKLEQGVEMLRIFKEHDARTSILDDFDFYDERERSLKERRAKQQVS
	orange1.1g006798m	CsiDF3B	NGTLDGILKGSYNQLDFVTNYKVAKFFIIKSYSEDNVHKSIKYGLWASTPNGNKKLDAAYREAKEKHG<br>TCPIFLLFSVNASAQFCGVAEMVGPVDFDKSVDYWLQDKWSGQFPVKWHIIKDVPNSQFRHIILEKND<br>NKPVTNSRDTQEVELEQGIEMLNIFKNYESYSSILDDFHFYEQRQKAMQERKAGQQAN
	orange1.1g005441m	CsiDF1C	VEGNIIICTEHYNKEDFQVDYVDAKFFVIKSYSEDDVHKSIKYGVWSSTSHGNKKLQSAYEDAQKLAAG<br>KPGGCPIFLFFSVNASSQFCGVAEMIGPVDFYKDMDFWQQDKWSGSFPVKWHIIKDVPNTSLRHIILRN<br>NENKPVTNSRDTQEIMHTEGLEMLKIFKNHPRDTSLFDDFMYYEKRQKFMQEERARLVFK
	orange1.1g008255m	CsiDF2C	EVLSPTVSRDQYNLPDFQVEYEKAKFYVIKSYSEDDIHKCIKYDVWSSTPNGNKKLDATFNEAEAKADE<br>TGTRCPIFLFFSVNGSGQFVGLAEMMGKVDFNKDMDFWQLDKWNGFFPVKWHVIKDVPNTLLRHITL<br>ENNENKPVTHSRDTQEIGLKQGLEMLKIFKSYSAKTSLLDDFNFYENKERSFHGKKSSKPAT
	orange1.1g048663m	CsiDF3C	DSISSLIWKDQYNLPDFRIKYDHALFFVIKSYSEDDIHKSIKYSVWSSTPNGNKKLDAAYEDAQSRIAEKG<br>SKCPVFLFFSVNASGQFCGVAEMIGRVDFNKNMDFWQQDKWNGYFPVKWHIIKDVPNPQLRHIILEN<br>NDKKPVTNSRDTQEVKFPQGIEILNIFKNYPSKTSILDDFDFYESRQKVMQEKKVRLSVS
	orange1.1g005338m	CsiDC1A	NRNATPLPQGISRYFIVKSCNRENLELSVQQGVWATQRSNEAKLNEAFDSAENVILIFSVNRTRHFQGC<br>AKMTSKIGGSVGGGNWKYAHGTAHYGRNFSVKWLKLCELSFHKTRHLRNPYNENLPVKISRDCQELE<br>PSIGEQLAALLYLEPDSELMAISVAAEAKREEEK
	orange1.1g015749m	CsiDC1B	STDNNKGKLYNTRYFIIKSLNHQNIQLSIEKEIWATQVMNEPILEEAFHNSGKVILIFSVNMSGFFQGYA<br>QMMSSVGWRRDNVWSQGNGKNNPWGRSFKVKWLRLNTLPFQKTLHLKNPLNDYKPVKISRDCQEL<br>PQDIGEALCHLLDGKDDVDGIQTSFHRDDLPAKR
	AT3G03950	ETC1	DVSAVDLQRYNGENFPESFVKAKFFVIKSYSEDDVHNCIKYGAWSSTPTGNKKLNAAYYEAKENSQEC<br>PVYLLFSVNASGQFVGLAEMVGPVDFNKTMEYWQQDKWIGCFPVKWHIIKDIPNSLLRHITLANNEN<br>KPVTNSRDTQEVNLEHGTKIIKIFKEYMSKTCILDDYKFYETRQKIIRDKKIKQKKQ
	At3g13460	ECT2	DNTCVVPDREQYNKEDFPVDYANAMFFIIKSYSEDDVHKSIKYNVWASTPNGNKKLAAAYQEAQQK<br>AGGCPIFLFFSVNASGQFVGLAEMTGPVDFNTNVEYWQQDKWTGSFPLKWHIVKDVPNSLLKHITLE<br>NNENKPVTNSRDTQEVKLEQGLKIVKIFKEHSSKTCILDDFSFYEVRQKTILEKKAKQTQK
	AT5G61020	ECT3	SEDVSLLDPKDYNKIDFPETYTEAKFYVIKSYSEDDIHKSIKYSVWSSTPNGNKKLDASYNEAKQKSDGC<br>PVFLLFSVNTSGQFVGLAEMVGPVDFNKTVEYWQQDKWIGCFPVKWHFVKDIPNSSLRHITLENNEN<br>KPVTNSRDTQEVKLEQGIKVIKIFKDHASKTCILDDFEFYENRQKIIQERKSKHLQI
	AT1G55500	ECT4	TVTCVLPDREECNRDDFPVEYKDAKFFIIKSYSEDDVHKSIKYNVWASTPNGNKKLDAAYQEAQQKSS<br>GCPVFLFFSVNASGQFIGLAEMKGPVDFNKNIEYWQQDKWTGSFPLKWHILKDVPNSLLKHITLEYNE<br>NKPVTNSRDTQEVKLEQGLKVVKIFKEHNSKTCILDDFSFYEARQKTILEKKAKQQQS
	AT3G13060	ECT5	NKGSAKEHEESNNADFVTDYTNAKLFIIKSYSEDNVHKSIKYNVWASTPNGNKKLDAAYREAKDEKE<br>PCPLFLLFSVNASSQFCGVAEMVGPVDFEKSVDYWQQDKWSGQFPVKWHIIKDVPNSQFRHIILENND<br>NKPVTNSRDTQEVKLEQGIEMLKIFKNYDADTSILDDFGFYEEREKIIQDRKARRQPS
Arabidopsis thaliana	AT1G27960	ECT9	SKMISYDRVDRFCQQELLSQFRDAKFFVIKSYSEDNVHKSIKHCVWASTKNGNKKLDAAYREAKKKDV<br>ACPVFLLFSVNASSQFCGVAEMVGPVDFNTSVEYWQQDRWSGHFPVQWLIVKDVPNSLFRHIIIESND<br>NKPVTNSRDTQEVGLEKGIEMLDIFISCEMRSSILDDFNFYEERQIAIQDRKARQRAV
	AT5G58190	ECT10	DSSTAGPNPSLYNHPEFVTDYKNAKFFIVKSFSEDNVHRSIKYNVWASTPHGNKKLDTAYRDAEKMG<br>GKCPIFLFFSVNASGQFCGVSEMVGPVDFEKDAGYWQQDRWSGQFPVKWHIVKDIPNNRFCHILLQN<br>NDNKPVTHSRDSQEVKLRQGIEMLRIFKEYEAHTSILDDFGYYDELEGQKVGEDGTRKKA
	AT3G17330	ECT6	AEGNIVINPDRYNKEDFSIEYSDARFFVIKSYSEDDVHKSIKYGVWSSTLNGNKKLQSVYEDAQRIATEK<br>SRECPIFLFFSVNSSGLFCGVAEMTGPVSFDRDMDFWQQDKWSGSFPVKWHIIKDVPNSYFRHIILHNN<br>ENKPVTNSRDTQEIILKQGLEVLKLFKHHAEKTSLLDDFMYYEDRQRLMQEERARLPFR
	At1g48110	ECT7	AEGNIVINPSQYNKEDLRIDYSNAKFFVIKSYSEDDVHKSIKYNVWSSTLHGNKKLQSAYEDAQRIATE<br>KSCECPIFLFFSVNASGLFCGMAEMTGPVSFDKDMDFWQQDKWSGSFPVKWHIIKDVPNSYFRHIILQ<br>NNENKPVTNSRDTQEIMLKQGLEVLKIFKDHMERTSLLDDFVYYESRQRVMQDERTRLPYR
	AT1G79270	ECT8	NGVGSVIRRDQYNLPSFQTKYEEAIFFVIKSYSEDDIHKSIKYNVWSSTLNGNKKLDSAYQESQKKAADK<br>SGKCPVFLFFSVNASGQFCGVAEMIGRVDYEKSMEFWQQDKWTGYFPVKWHIIKDVPNPQLRHIILEN

			NENKPVTNSRDTQEVRLPQGNEVLNIFKNYAAKTSILDDFDFYENREKVMVQKKLRFPPV
	AT1G09810	ECT11	NSFALALRREMYNLPDFQTDYEDAKFFVIKSYSEDDVHKSIKYSVWSSTINGNKKLDAAFRDAETKTLEDGKKRPIFLFFSVNASRQFVGLAEMVGYVDFNKDLDFWQVDKWSGFFPVEWHVVKDIPNWELRHIILDNNEDKPVTHTRDTHEIKLKEGLQMLSIFKKYSAVTFLLDDMDFYEEREKSLRAKKEHKPAT
	AT1G30460	CPSF30	NRTSHPLPQGVNRYFVVKSNNRENFELSVQQGVWATQRSNEAKLNEAFDSVENVILIFSVNRTRHFQGCAKMTSRIGGYIGGGNWKHEHGTAQYGRNFSVKWLKLCELSFHKTRNLRNPYNENLPVKISRDCQELEPSVGEQLASLLYLEPDSELMAISIAAEAKREEEK
	AT4G11970	ECT12	KANKNSKPGYRTRYFIIKSLNYDNIQVSVEKGIWATQVMNEPILEGAFHKSGRVILIFSVNMSGFFQGYAEMLSPVGWRRDQIWSQGGGKNNPWGRSFKVKWLRLSELPFQKTLHLKNPLNDYKPVKISRDCQELPEDIGEALCELLDANSCDDGLLNSSSRDDYSTKR
Camellia chekiangoleosa	Cch01T002587.1	CchYTH1	NAKFFVIKSYSEDDVHKGIKYNVWSSTPNGNKKLQSAYEDAQRIASGEPRGCAIFLFFSVSDIPLILSFALKYYIPQVNASGQFCGVAEMTGPVDFHKDMDFWQQDKWSGSFPVKWHIIKDVPNPNFRHIILENNENKPVTNSRDTQEIRCRQGIEMLKIFKLYTSK
	Cch02T002736.1	CchYTH2	NAKFFVIKSYSEDDVHKGIKYNVWSSTPNGNKKLRSAYEDAQRIAFGEPRGCPIFLFFSVRDIPLILSFALKYYIPQVNASGQFCGVAEMTGPVDFHKDMDFWQQDKWSGSFPVKWHIIKDVPNPNFRHIILENNENKPVTNSRDTQEMFFSTHYAGDNLTIKLRD
	Cch02T003694.1	CchYTH3	DAKFFIIKSYSEDNVHKSIKYGIWASTPNGNRKLDAAYREAKEKNTCPVFLFFSVNASAQFCGVAEMVGPVDFDKSVDYWQQDKWSGQFPVKWHIIRDVPNSQFRHIVLENNDNKPVTNSRDTQEAKLEQGIEMLKIFNNYES
	Cch03T002158.1	CchYTH4	NAKFFIIKSFSEDNVHKSIKYSVWASTPLGNRKLDAAYHEVKDANGHCPVFLLFSVNASGQFCGVAEMIGPVDFEHDVDYWQQDRWSGQFPVRWHIIKDVPNNQFRHILLENNDNKPVTHSRDSQEVKLEQGIEILKIFKDYEA
	Cch05T000069.1	CchYTH5	NAKFFVIKSYSEDDVHKSIKYNVWSSTPNGNKKLQSAYEDARRIAAGEPRGCPVFLFFSVNASGQFCGVAEMTGPVDFYKDMDFWQQDKWSGSFPVKWHIIKDVPNPNFRHIILENNENKPVTNSRDTQEVCFVFIPDTLFTC
	Cch07T000910.1	CchYTH6	NAKFYVIKSYSEDDIHKCIKYDVWSSTPNGNKKLDAAFHDAEAKACETGTKCPIFLFFSVNGSGQFIGVAEMIGPVDFNKDMDFWQLDKWNGFFPVKWHIIKDTPNTQLRHIILENNDNRSVTYSRDTQEIGFKQGIEMLNIFNSYSE
	Cch08T001554.1	CchYTH7	ISRYFIVKSCNRENLELSVEQGVWATQRSNEAKLNEAFDSVENVILIFSVNRTRHFQGCAKMTSKIGGSVGGGNWKYAHGSAHYGRNFSVKWLKLCELSFHKTRLLRNPYNENLPVKISRDCQELEPSVGEQLASLLYLEPD
	Cch09T004551.1	CchYTH8	NAKFFVIKSYSEDDVHKGIKYNVWSSTPNGNKKLQSAYEDAQRIASGEPRGCAIFLFFSVSDIPLILSFALKYYIPQVNASGQFCGVAEMTGPVDFHKDMDFWQQDKWSGSFPVKWHIIKDVPNPNFRHIILENNENKPVTNSRDTQEIRCRQGIEMLKIFKLYTS
	Cch12T001174.1	CchYTH9	DAKFFIIKSYSEDDVHKSIKYNVWASTPNGNKKLDAAYQEAQQKSGGCPVFLFFSVNTSGQFVGVAEMTGPVDFHKNFEYWQQDKWNGCFPVNWHIVKDVSNSLLKHITLENNENKPVTNSRDTQEVKLEQGLQLLKIFKDHSSK
	Cch13T001809.1	CchYTH10	GTRYFIIKSLTHQNIQLSIEKGIWATQVMNEPILEEAFHNSSKVILIFSVNMSGFFQGYAQMMSSVGWRRDNVWSQGSGGKNPWGRSFKVKWLRLHDLPFQKTLHLKNPLNDYKPVKISRDCQELPQDIGEALCELLDGKDD

* All accession numbers relate to the JGI Phytozome, Metazome or MycoCosm databases
** In cases when a name was already attributed to a protein, this name was kept with the new name, refering to the species of origin and to the phylogenetic classification of the protein, added in parenthesis
*** For these species, only the core YTH motif are indicated

Table S2. Conserved Motif analysis of YTH proteins in *Camellia chekiangoleosa*.

Motif	Logo	E-value	Sites	Width
1	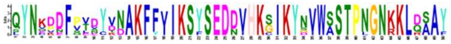	1.3e-218	8	50
2	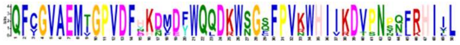	4.9e-215	7	50
3	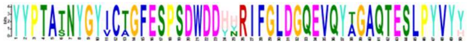	1.2e-096	4	50
4	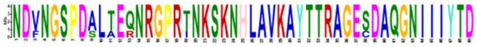	7.3e-088	4	49
5	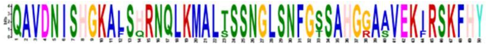	2.3e-084	4	50
6	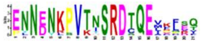	4.6e-084	10	21
7	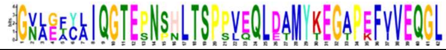	1.6e-052	4	42

8	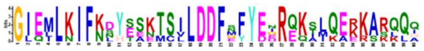	6.5e-050	5	40
9	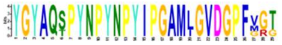	4.1e-045	4	29
10	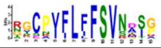	1.7e-043	10	15

Table S3. Primer sequences.

Primer Name	Primer Sequense (5'-3')
CchYTH1-Q-F	GGTGCTATGTTAGGAGTTG
CchYTH1-Q-R	AAGCCATTACTAGAAGTGAGA
CchYTH2-Q-F	CAAGAACGGTACAATCAGC
CchYTH2-Q-R	TACACGAAACTCACCAAAT
CchYTH3-Q-F	ATGAGTCTTACAACTGCCCGAA
CchYTH3-Q-R	GTGTGCTGGCCCATATACCAT
CchYTH4-Q-F	AGTCTTTCTCTTGTTTTCGGTCA
CchYTH4-Q-R	CTTTGATTATATGCCACCGGACA
CchYTH5-Q-F	CCCTTCTTAGATACTGGTGCAT
CchYTH5-Q-R	TACCCTAGCAAAGGAACGTGTC
CchYTH6-Q-F	GCAAGCTTCATCTCAAACCGAA
CchYTH6-Q-R	TGCTGCTTCTTCAGTTGTCGTT
CchYTH7-Q-F	CGGCTAACGACAAGAACGA
CchYTH7-Q-R	TCATCAGCTCCCGAACCAC
CchYTH8-Q-F	GACGCTGTTGTAGGTAATG
CchYTH8-Q-R	GATGTAGCTGATCGGAGAT
CchYTH9-Q-F	CGTCCTCATTCTCATCTCA
CchYTH9-Q-R	TCTAAAGGTGCTTCCATAC
CchYTH10-Q-F	TCTAAAGGTGCTTCCATAC
CchYTH10-Q-R	CGCAGCCATTTCACCTTAA
Actin-F	GTGGTTGTGAATGGAGGGCA
Actin-R	AGGCCAGGAGACCCATTACA
CchYTH9-F	ATGGTTTCATCAATATCACATGGAAATAACTCCCC
CchYTH9-R	CATGGGAAAAAATGTACCCTCAACATTTGCAA
1305-CchYTH9-F	TTTGGAGAGAACACGTCTAGAATGGTTTCATCAATATCACATGGAA
1305-CchYTH9-R	CTTGCTCACCATGTAGTCGACCATGGGAAAAAATGTACCCT

Table S4. PLAAC of YTH proteins in *Camellia chekiangoleosa*.

Protein Name	LLR	PAPAprop	PAPAfi
CchYTH1	1.709	-0.043	-0.126
CchYTH2	7.976	0.011	-0.005
CchYTH3	8.537	-0.022	-0.004
CchYTH4	5.516	0.047	-0.043

CchYTH5	10.072	0.023	-0.002
CchYTH6	10.361	0.109	-0.096
CchYTH7	23.847	0.019	-0.173
CchYTH8	6.537	-0.007	-0.005
CchYTH9	11.980	0.016	-0.168
CchYTH10	-11.558	0.053	-0.007

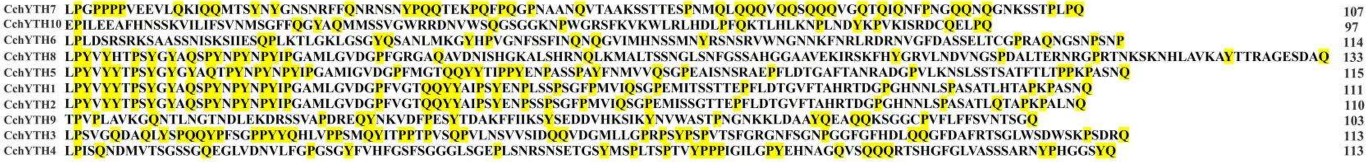

Figure S1. Y/P/Q-rich region in CchYTHs. The Y/P/Q amino acids are highlighted in yellow. The number at the end represents the number of amino acids.
